# Lack of Acid Sphingomyelinase Induces Age-Related Retinal Degeneration

**DOI:** 10.1371/journal.pone.0133032

**Published:** 2015-07-13

**Authors:** Bill X. Wu, Jie Fan, Nicholas P. Boyer, Russell W. Jenkins, Yiannis Koutalos, Yusuf A. Hannun, Craig E. Crosson

**Affiliations:** 1 Department of Biochemistry and Molecular Biology, Medical University of South Carolina, Charleston, South Carolina, United States of America; 2 Department of Ophthalmology, Storm Eye Institute, Medical University of South Carolina, Charleston, South Carolina, United States of America; 3 Stony Brook Cancer Center and Department of Medicine, Stony Brook University, 100 Nicolls Rd., Stony Brook, New York, United States of America; University of Cologne, GERMANY

## Abstract

**Background:**

Mutations of acid sphingomyelinase (ASMase) cause Niemann–Pick diseases type A and B, which are fatal inherited lipid lysosomal storage diseases, characterized with visceral organ abnormalities and neurodegeneration. However, the effects of suppressing retinal ASMase expression are not understood. The goal of this study was to determine if the disruption of ASMase expression impacts the retinal structure and function in the mouse, and begin to investigate the mechanisms underlying these abnormalities.

**Methods:**

Acid sphingomyelinase knockout (ASMase KO) mice were utilized to study the roles of this sphingolipid metabolizing enzyme in the retina. Electroretinogram and morphometric analysis were used to assess the retinal function and structure at various ages. Sphingolipid profile was determined by liquid chromatography-mass spectrometry. Western blots evaluated the level of the autophagy marker LC3-II.

**Results:**

When compared to control animals, ASMase KO mice exhibited significant age-dependent reduction in ERG a- and b-wave amplitudes. Associated with these functional deficits, morphometric analysis revealed progressive thinning of retinal layers; however, the most prominent degeneration was observed in the photoreceptor and outer nuclear layer. Additional analyses of ASMase KO mice revealed early reduction in ERG c-wave amplitudes and increased lipofuscin accumulation in the retinal pigment epithelium (RPE). Sphingolipid analyses showed abnormal accumulation of sphingomyelin and sphingosine in ASMase KO retinas. Western blot analyses showed a higher level of the autophagosome marker LC3-II.

**Conclusions:**

These studies demonstrate that ASMase is necessary for the maintenance of normal retinal structure and function. The early outer retinal dysfunction, outer segment degeneration, accumulation of lipofuscin and autophagosome markers provide evidence that disruption of lysosomal function contributes to the age-dependent retinal degeneration exhibited by ASMase KO mice.

## Introduction

Lipid metabolism is essential for maintaining the structure and function of the visual system. Evidence has shown that chronic misregulation of retinal lipid metabolism can result in retinal degeneration and blindness [1]. Importantly, specific mutations of lipid metabolizing proteins have also been linked to retinal degeneration. These examples include mutations in hepatic lipase gene, the ATP-binding cassette transporters 1, and lysophosphatidylcholine acyltransferase 1 which all lead to the retinal degeneration [2–4]. Conversely, lipids also play important prosurvival roles in the retina. Docosahexaenoic acid has been shown to protect photoreceptors and retinal pigment epithelium (RPE) cells from oxidative damage [5, 6], and the bioactive lipid product inositol-1, 4, 5-trisphosphate is an important signaling molecule involved in retinal neuroprotection [7]. While these studies have extended our knowledge regarding the roles of lipids in visual function and pathology, many gaps still remain.

Sphingolipids (SPL) represent a major class of lipids that form important components of cellular membranes, and many studies have shown that SPL play important roles in regulating diverse cellular events, such as cell growth arrest, senescence, apoptosis, and inflammation and degeneration [8, 9]. Additionally, studies have provided evidence that misregulation of SPL metabolism can lead to retinal degeneration (review [10, 11]). Importantly, some lipid storage diseases, which are caused by disorders of sphingolipid metabolism, such as Krabbe’s disease [12], and Sandhoff disease [13], are characterized by vision loss. Although current evidence suggests a strong connection between SPL metabolism and retinopathy, the exact roles of these lipids in the retina are mostly unknown.

Among various SPL regulating enzymes, acid sphingomyelinase (ASMase) plays an essential role in regulating SPL metabolism by hydrolyzing the phosphodiester bond of sphingomyelin (SM), yielding ceramide, the “hub” of SPL metabolism [14]. Mutations of ASMase result in Niemann-Pick disease (NPD) types A and B, a fatal autosomal recessive lysosomal storage disorder, presenting both visceral and neurological symptoms. ASMase knockout (KO) mice have been generated, providing the animal model for NPD types A and B diseases [15, 16]. In the ASMase KO mice, neuronal degeneration has been identified in the brain, leading to the dysfunction of neuromotor coordination. Progressive degeneration of specific neuronal cell types, specifically cerebellar Purkinje cells, was also identified [17]. However, the molecular mechanism of neurodegeneration in NPD patients is still obscure [18] and the impact on other organ systems has been barely investigated.

In the eye, previous studies have provided only limited and conflicting information regarding the impact of ASMase dysregulation on retinal structure and function [19–23]. As a result the current study was conducted to begin to understand the relationship between sphingolipid metabolism and visual impairments. Morphometric and electroretinogram (ERG) responses and analysis were used to assess age-dependent changes in retinal structure and function in ASMase KO mice. Our results demonstrate that ASMase is necessary for the maintenance of normal retinal structure and visual function and that the deletion of ASMase results in progressive retinal degeneration.

## Materials and Methods

### Animals

ASMase KO mice with C57/BL6 background were the generous gift of Dr. Edward H. Schuchman (Icahn Medical Institute). The heterozygous mice (SMPD1^+/-^) were bred, and the mice were genotyped as previously described [15]. The homozygous and wild-type (WT) littermates were used for experiments. All experiments were performed in strict accordance with the ARVO Statement for the Use of Animals in Ophthalmic and Vision Research; and the study protocol was approved by the Medical University of South Carolina Animal Care and Use Committee (Approval Number: 2947 and 2914).

### Cell Culture

Control dermal fibroblasts (from the unaffected mother of a child with Lesch-Nyhan, GM02226) and Niemann-Pick type A dermal fibroblasts (NPD, GM13205) were obtained from the Coriell Cell Repository (Camden, NJ), which is part of NIH cell repository and provides scientists with resources for cell and genetic research. These two cell lines have been used in the previous publication[24]. Cells were maintained in high-glucose Dulbecco Minimum Essential Medium (Invitrogen) supplemented with 10% (v/v) fetal bovine serum (Invitrogen).

### *In vitro* ASMase Activity Assay

*In vitro* ASMase enzymatic assays were performed as described previously with modifications [25]. Briefly, various tissues were collected from C57/BL6 mice and minced with scissors in lysis buffer (0.2% Triton X-100, 50 mM Tris-HCl, pH 7.4, protease inhibitor cocktail (Roche, Germany)). The tissues were then sonicated briefly, and tissue debris and unbroken cells were pelleted and removed by centrifugation at 800 x *g* for 5 min at 4°C. Each reaction mixture contains 1 mM EDTA, 250 mM sodium acetate, 100 mM choline-methyl-^14^C sphingomyelin (Lipidomics Core Facility, at the Medical University of South Carolina, Charleston, SC), and 0.2% Triton X-100, pH 5.0. After incubation for 0.5 h at 37°C, the reaction was stopped by adding 1.5 mL of chloroform/methanol (2:1; Sigma Aldrich, St. Louis, MO), followed by adding 400 μL of water. Phases were separated by centrifugation at 2000 x *g* for 5 min. The upper phase was subjected to scintillation counting. ASMase activity was determined by quantification of the amount of the released radioactive phosphocholine.

### Electroretinogram (ERG) Recordings

The ERG recordings were performed as described previously [26]. Briefly, overnight dark-adapted mice were anesthetized using xylazine (20 mg/kg, i.p.) and ketamine (80 mg/kg, i.p.). Pupils were dilated with phenylephrine hydrochloride (2.5%) and atropine sulfate (1%). Contact-lens electrodes were placed on both eyes accompanied by methylcellulose. Full-field ERGs were recorded using the universal testing and electrophysiologic system 2000 (UTAS E-2000, LKC Technologies Inc., Gaithersburg, MD). Single flashes of 10 ms duration with intensities of 2.48 cd*s/m^2^ were used for stimulation under scotopic conditions. Rod function was recorded under scotopic conditions. M- and S-cone functions were recorded under photopic conditions using green and UV light. RPE function was evaluated by the c-wave recordings with 100 cd/m^2^ stimulus.

### Histology

Eyes were enucleated and immersion-fixed in a solution of 30% chloroform, 60% methanol, and 10% acetic acid at 4°C overnight, and dehydrated over several hours before being embedded in paraffin in transverse orientation. Eyes were sectioned (7 μm thick) through the optic nerve heads and mounted on poly-L-lysine-coated slides. The sections were stained with hematoxylin and eosin, dehydrated and coverslipped with mounting medium (Permount; Fisher Scientific, Fair Lawn, NJ). Central areas of the retina (within 100 to 300 μm of the optic nerve) were photographed for documentation. Images were acquired on a Zeiss microscope (Axioplan 2, Germany).

### Western Blot Analysis

The eyecups or cells were homogenized in lysis buffer (25 mM Tris, 1 mM EDTA, 1 x protease inhibitor cocktail [Roche], pH 7.4) by brief sonication. Equal amount of total proteins from each lysate were loaded onto polyacrylamide gels and subjected to Western blot analysis using primary antibodies followed by horseradish-peroxidase-labeled secondary antibody (1:5000 dilutions, Santa Cruz Biotechnology, Inc. Dallas, TX). The signals were detected using ECL reagents (Thermo Scientific, Waltham, MA). The antibodies against LC-3 (1:1000 dilutions) were from MBL International Corporation (Woburn, MA). The Beclin-1 antibody (1:1000 dilutions) was purchased from Cell Signaling Technology (Danvers, MA). β-actin antibody (1:30,000 dilution) was purchased from Sigma Aldrich (St. Louis, MO). Cells were treated with pepstatin A (10 *μ*g/ml, Sigma Aldrich, St. Louis, MO) and E-64d (10 *μ*g/ml, Sigma Aldrich, St. Louis, MO) for 24 h.

### RPE Lipofuscin

Published procedures [27] were followed. Enucleated eyes were hemisected and the anterior segment, lens and vitreous were removed. The retina was peeled off and 4–5 shallow incisions were made to allow flattening of the posterior eyecup. The flattened posterior eyecups were mounted freshly on microcope slides and covered gently with a coverslip. Color images of RPE fluorescence were captured on a Zeiss Axioplan II epifluorescence microscope (Carl Zeiss, Thornwood, NY) with a 63× oil immersion objective lens and a Nikon D (Nikon, INC., Melville, NY) digital camera. Fluorescence was excited with 450–490nm light, and the emission was collected > 510nm. Fluorescence emission spectra of RPE granules were acquired on an SP2 Leica laser scanning confocal microscope (Leica Microsystems, Inc., Buffalo Grove, IL) with a 63× oil immersion lens using 488nm excitation. Spectra were corrected for background by subtracting the spectrum of a region from the same field that did not contain granules. For normalization, the average granule spectra were divided by the value of the maximum (average) intensity.

### LC/MS Analysis of Endogenous Sphingolipids

Sphingolipid analyses were performed by the Lipidomics Core Facility at the Medical University of South Carolina, using Electrospray Ionization/Tandem Mass Spectrometry (ESI-MS/MS) on a Thermo Finnigan TSQ 7000 triple-stage quadrupole mass spectrometer, operating in a multiple reaction monitoring positive ionization mode [28].

### Statistical Analysis

Results are presented as the mean ± SE unless otherwise stated. Comparisons among groups were made by two-tailed Student *t* tests, accepting a significance level of *P* < 0.05.

## Results

### Comparison of ASMase activity between retina and other tissues

**Fig 1:**
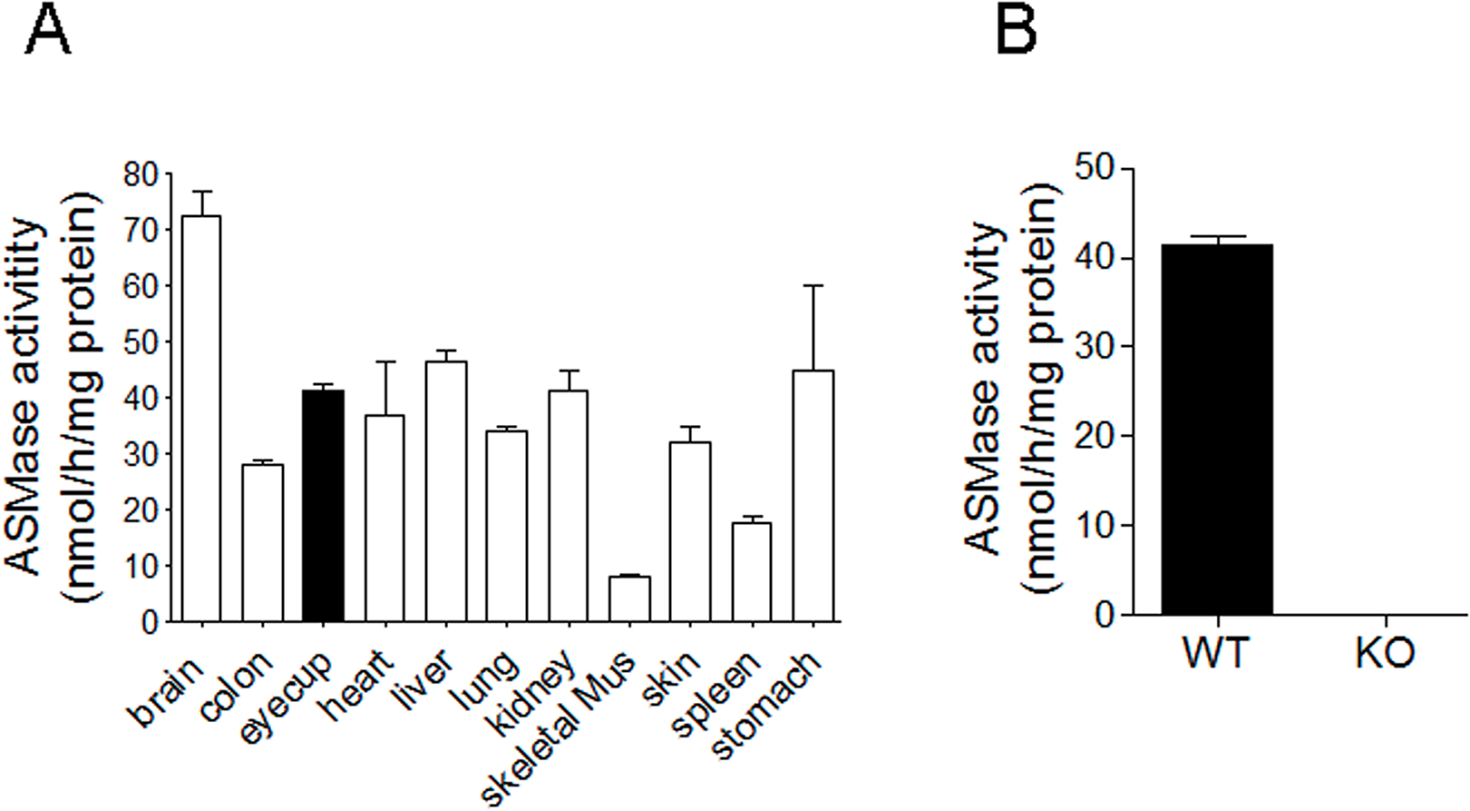
ASMase activities in mouse tissues. (**A**) ASMase activities in mouse eyecups (black bar) and other tissues (white bars) including brain, colon, heart, liver, lung, kidney, skeleton muscle, skin, spleen and stomach; (**B**) ASMase activities in WT and ASMase KO eyecups. Data are expressed as mean ± SE; n = 3.

To evaluate the retinal ASMase activity, we compared the ASMase activities in the posterior eyecup to activities in the brain and other major organs. As shown in Fig 1, the highest ASMase activity was measured in the brain. The ASMase activity measured in the posterior eyecup was approximately equal to that measured in the heart, lung, kidney and stomach, whereas skin, skeletal muscle and spleen had lower activity. However, as the eyecup preparation contained the posterior sclera (connective tissue), our estimates likely underestimate the specific activity of the enzyme in the retina. Although multiple sphingomyelinases exist, deletion of ASMase resulted in a complete loss of ASMase activity in the posterior eyecup (Fig 1B) and in various tissue [15, 16], confirming that under these conditions, the assay detected exclusively ASMase.

### Age-dependent neural retinal changes in ASMase KO mice

**Fig 2:**
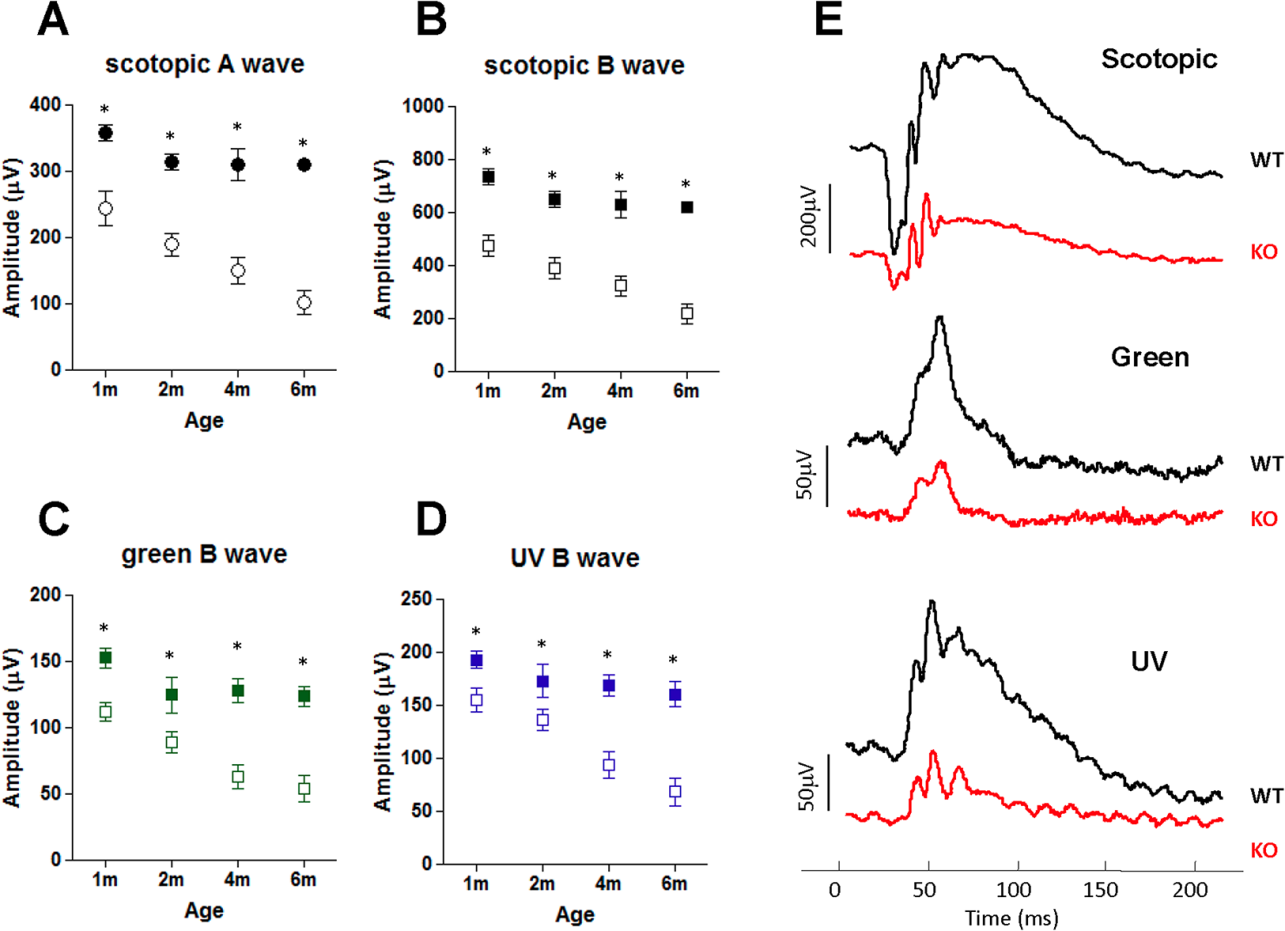
Effect of deletion of ASMase on electroretinogram responses. (**A**) Data analyses of scotopic ERG a-wave amplitudes from 1-, 2-, 4- and 6-month-old WT (filled circle) and KO (open circle) mice; (**B**) data analyses of scotopic ERG b-wave amplitudes from 1-, 2-, 4- and 6-month-old WT (filled square) and KO (open square) mice. (**C**) Data analyses of photopic ERG b-wave amplitudes for S cones and (**D**) M cones from 1-, 2-, 4- and 6-month-old WT (filled squares) and KO (open squares) mice. (**E**) Representative scotopic and photopic ERG signals in 6-month-old WT (black line) and KO (red line) mice. Each ERG was obtained by averaging two responses to 2.48 cd∙s/m^2^ flashes with an interstimulus interval of 60 seconds. Data are expressed as mean ± SE; *n* ≥6 (WT), ≥4 (KO) mice. *Indicates significant difference (*P* < 0.05) between responses in WT and ASMase KO mice.

To investigate if ASMase is necessary in the development and maintenance of neural retinal function *in vivo*, scotopic (dark-adapted) and photopic (light-adapted) ERG responses were measured in age-matched ASMase KO and WT littermates between 1 and 6 months-of-age (Fig 2). Comparison of scotopic ERGs in ASMase KO mice and WT mice demonstrated that by 1 month-of-age a- and b-wave amplitudes in KO mice were significantly reduced by 32 ± 6.7% and 35 ± 5.1%, respectively. Between the 2 and 6 months-of-age ERGs, analyses showed progressive reductions in both a- and b-wave amplitudes. Comparing ASMase KO mice to age-matched WT mice at 6 months demonstrated that a- and b-waves were significantly reduced by 67 ± 5.9% and 64 ± 6.1%, respectively. Analysis of photopic ERGs using UV and green light stimuli to evaluate the S and M cone functions also demonstrated significant age-dependent reductions in a- and b-wave amplitudes in ASMase KO mice when compared to WT mice (Fig 2C and 2D).

**Fig 3:**
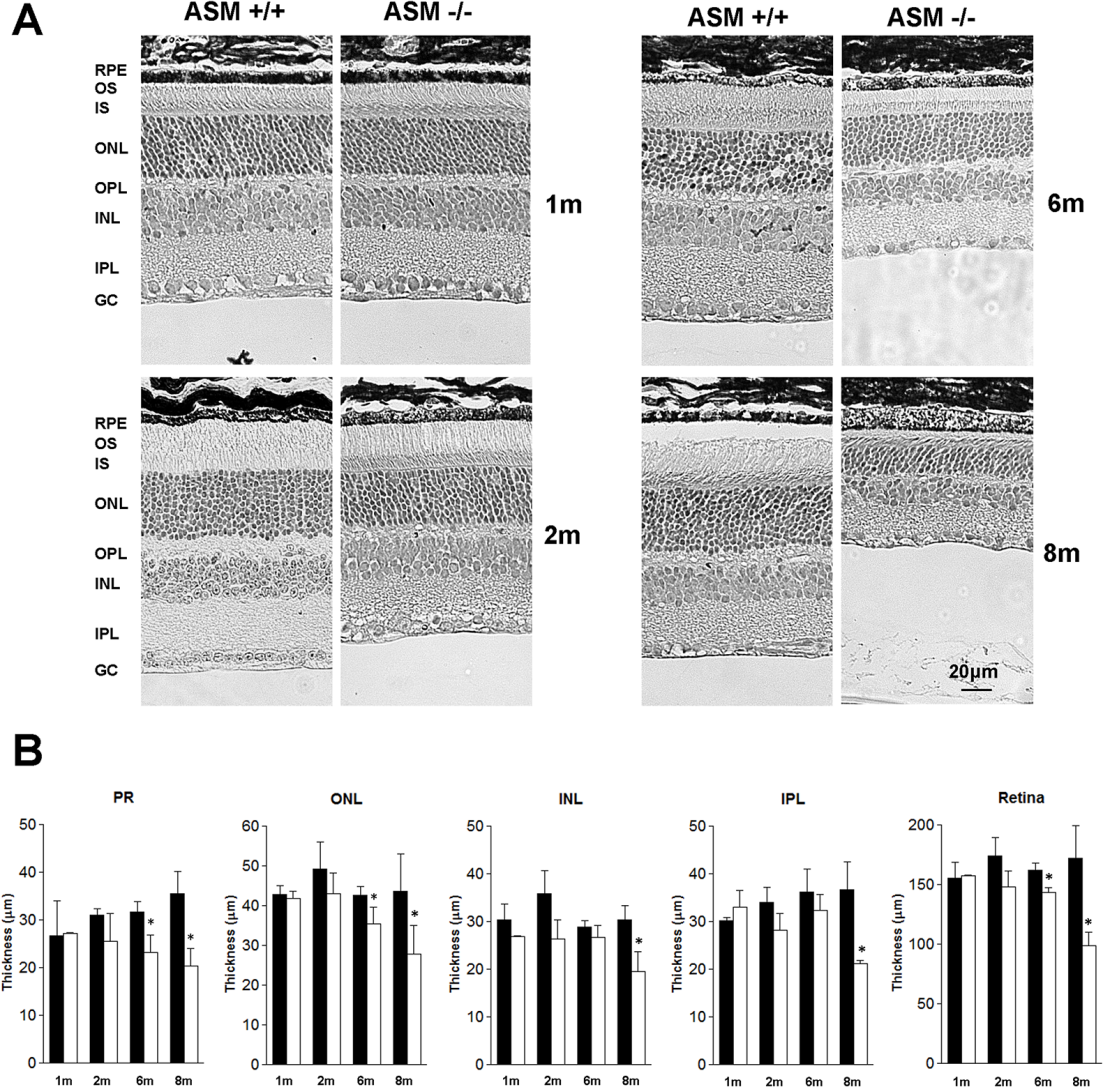
Effect of deletion of ASMase on retinal morphology. (**A**)Photomicrograph of retina cross-section in WT and ASMase KO mice at 1-, 2-, 6- and 8-months-of-age. All images were taken 2 to 3 disc diameters from the optic nerve. Scale bar is 20 μm. Abbreviations: retinal pigment epithelium (RPE); outer segment (OS); inner segment (IS); outer nuclear layer (ONL); outer plexiform layer (OPL); inner nuclear layer (INL); inner plexiform layer (IPL) and ganglion cell layer (GCL). (**B**) Morphometric analyses of the retinas from ASMase KO and WT mice. Data are expressed as mean ± SE; *n* ≥3 (WT), ≥4 (KO) mice. *Indicates significant difference (*P* < 0.05) between responses in WT and ASMase KO mice.

To examine the effect of ASMase on retinal structure, retinal cross-sections were evaluated in age-matched ASMase KO and WT mice from 1 to 8 months-of-age (Fig 3). In WT mice from1 to 8 months-of-age, morphometric analysis of the retina or individual retinal layers found no significant differences in the thickness. Comparing retinal analysis of WT and KO mice at 1 and 2 months-of-age found no significant difference in the thickness of the retina or individual retinal layers. However, at 6 months-of-age comparing ASMase KO mice to WT mice significant decreases in the photoreceptor and outer nuclear layer and total retinal thickness were measured. At 8 months-of-age progressive thinning of photoreceptor, outer nuclear layers were measured, as well as significant thinning of the inner nuclear and inner plexiform layers were measured. As a result of this degeneration, mean retinal thickness in ASMase KO mice, at 8 months-of-age was 36.8± 6.5% less than that measured in age match WT mice.

### Retinal pigment epithelial changes in ASMase KO mice

**Fig 4:**
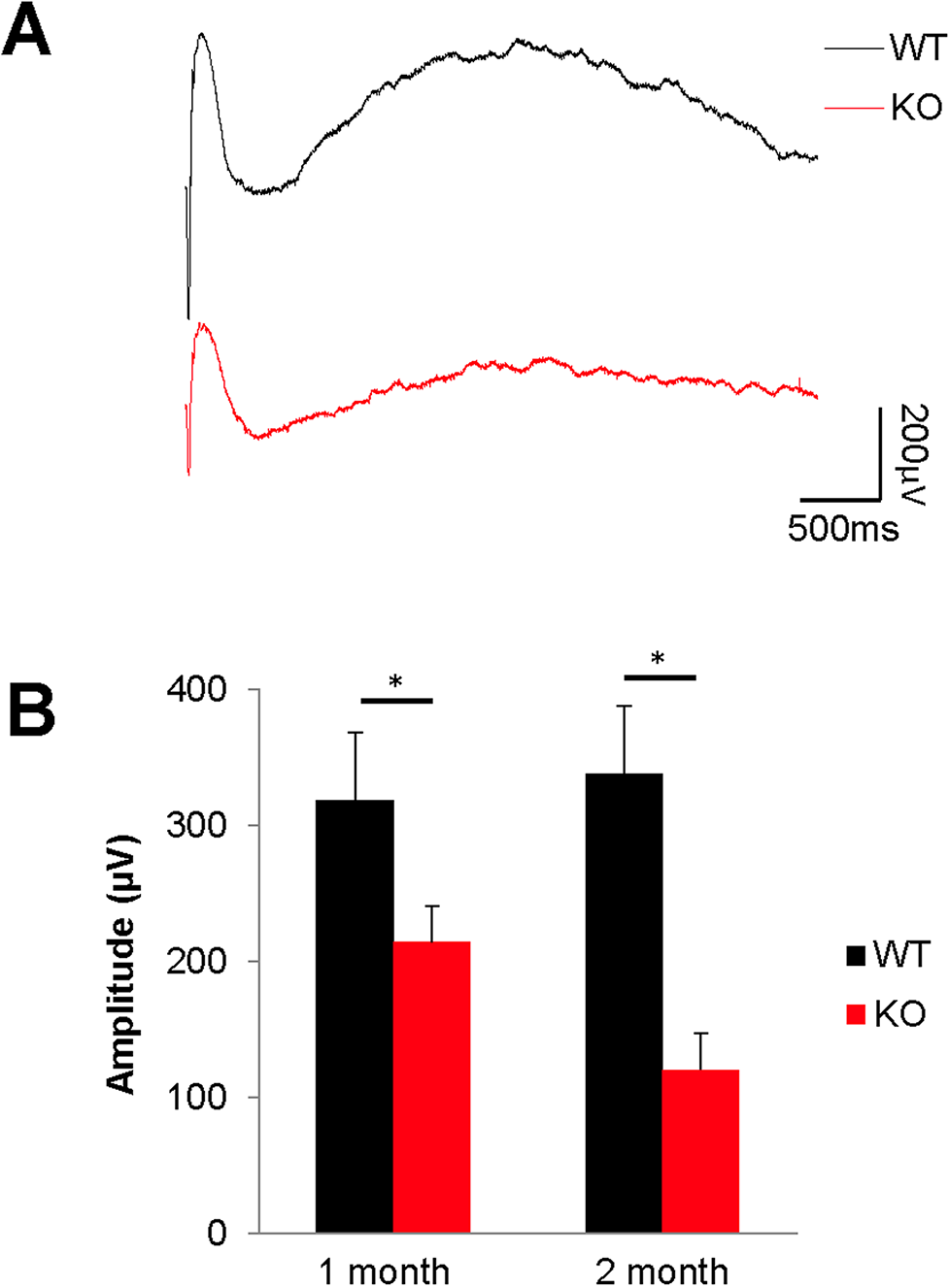
Effect of deletion of ASMase on RPE. (**A**) Representative c-waves in 2-month-old WT (black) and KO (red) mice. (**B**) Data analyses of c-wave amplitudes in WT (black bars) and KO (red bars) mice at 1 month and 2 months-of-age. Data are presented as mean ± SE, *n* = 8. *Indicates significant difference (*P* < 0.05) between responses in WT and ASMase KO mice.

As functional deficits in photoreceptors (i.e., decreased a-wave amplitudes) were evident by 1 month, early changes in RPE function and structure were evaluated. To investigate if the disruption of ASMase affects the RPE function, we evaluated the ERG c-wave amplitudes and autofluorescence signals. As shown in Fig 4, at 1 month-of-age, the mean c-wave amplitude in ASMase KO mice (213.9 ± 26.3 μV) was significantly lower than that in WT mice (318.2 ± 14.2 μV), and amplitudes continued to decline to 120.2 ± 26.8 μV at 2 months-of- age. No significant difference of c-wave amplitudes was detected between 1 and 2 months old WT mice.

**Fig 5:**
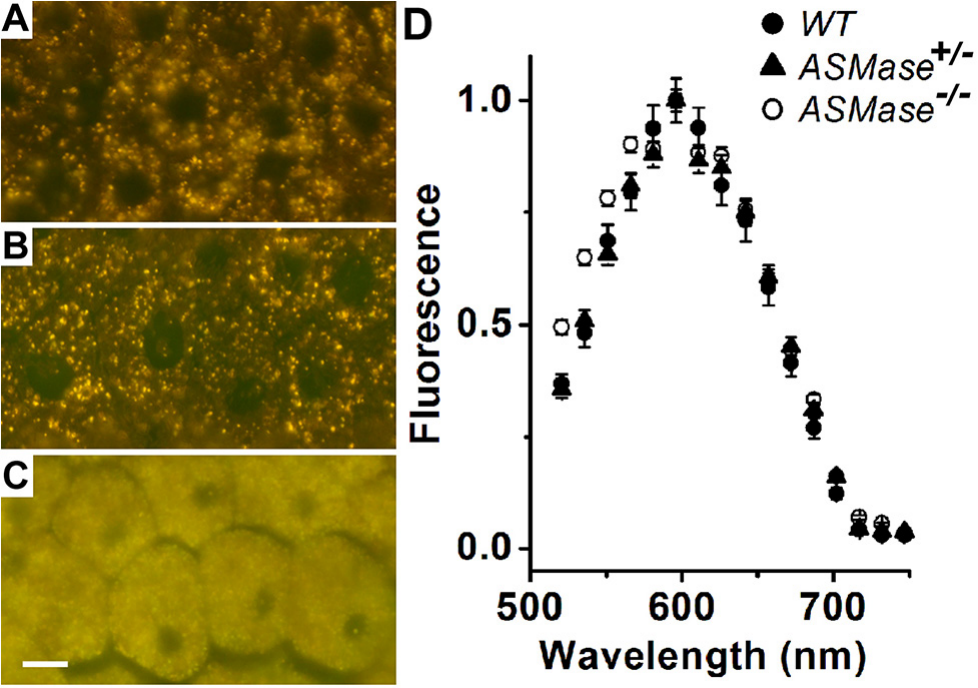
RPE lipofuscin of ASMase KO mice. True color fluorescence micrographs (excitation 450–490nm) of flat-mounted RPEs from 6-month-old (**A**) ASMase^+/+^, (**B**) ASMase^+/-^, (**C**) ASMase^-/-^. Scale bar is 10μm; (**D**) Emission spectra of the granule autofluorescence (excitation 488 nm) from ASMase^+/+^ (n = 72), ASMase^+/-^ (n = 69) and ASMase^-/-^ (n = 49) granules. Error bars represent S.E.

In the eye, the RPE is responsible for the daily phagocytosis of photoreceptor outer segments, a process coupled to their daily renewal. Phagocytosed outer segment material enters the RPE lysosomal compartment where it is degraded. Undigested material accumulates in the form of lipofuscin granules. Excessive accumulation of lipofuscin is associated with retinal degeneration [29, 30]. We therefore examined lipofuscin accumulation in the RPE of WT, ASMase heterozygous knockout (ASMase^+/-^) and ASMase homozygous knockout (ASMase^-/-^) mice at 6 months-of- age (Fig 5A–5C). RPE cells from 6-month-old WT and ASMase^+/-^ mice show significant accumulation of lipofuscin granules (Fig 5A and 5B). There is extensive accumulation of lipofuscin granules in the RPE cells from 6-month-old ASMase KO mice to the extent of filling up the cytoplasm (Fig 5C). The fluorescence emission spectra (excitation 488 nm) of individual granules from WT (n = 72), ASMase^+/-^ (n = 69), and ASMase KO (n = 49) were broadly similar, with a peak at ~600 nm (Fig 5D). The emission spectrum is characteristic of the presence of by-products of vitamin A metabolism [27], such as bis-retinoids [31, 32].

### Retinal sphingolipid levels in ASMase KO and WT mice

**Fig 6:**
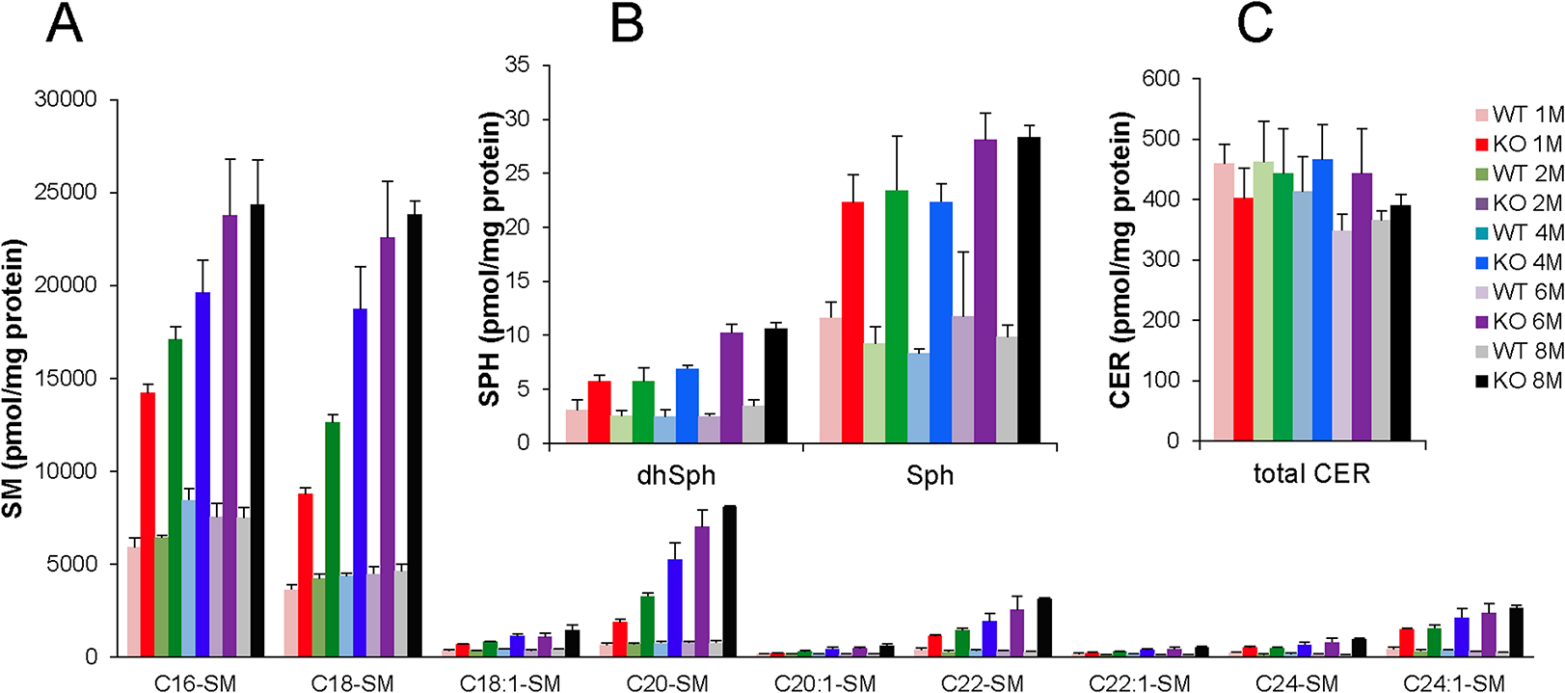
Effect of deletion ASMase on SPL profiles. (**A**) Sphingomyelin (SM) profile and (**B**) dihydrosphingosine and sphingosine profiles and (**C**) ceramide profile in WT (lighter color bars) and ASMase KO (darker color bars) retinas from 1-, 2-, 4-, 6- and 8-month-old mice. Data are presented as mean ± SD, *n* = 3.

As shown in Fig 6, the deletion of ASMase significantly increased SM levels in the retina at various ages (Fig 6A). In addition, several very long chain SMs (C20, C22, C22:1, C24:1, C26, C26:1) displayed large age-dependent increases. Interestingly, sphingosine and dihydrosphingosine levels were also elevated in the ASMase KO mice in an age-dependent manner (Fig 6B). However, no significant changes in the ceramide level were observed between ASMase KO and WT retinas (Fig 6C). Retinal S1P levels were below the level of detection.

### Autophagy dysfunction in ASMase KO eyecups

**Fig 7:**
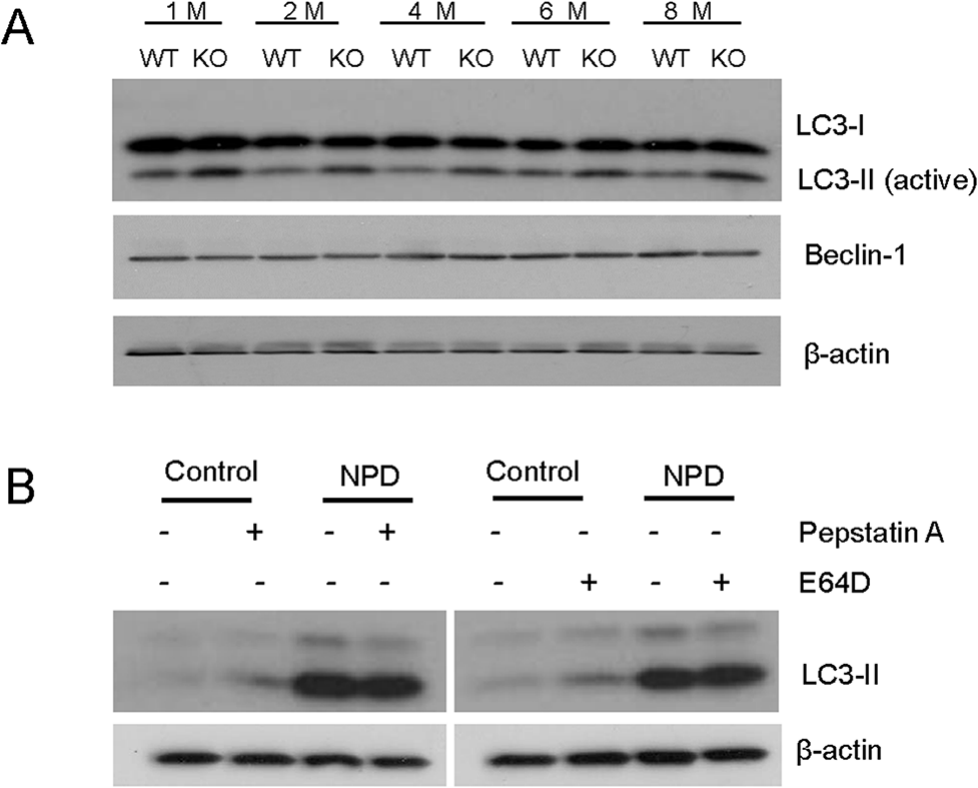
Effect of the deletion of ASMase on LC3 levels. (**A**) Expression levels of LC3-I, LC3-II and beclin-1 in WT and ASMase KO eyecups from 1-, 2-, 4-, 6- and 8-month-old mice. Beta-actin was used for loading controls. (**B**) Expression of LC3-II levels in control fibroblast cell line (Control) and Niemann-Pick type A dermal fibroblast cell line (NPD) with and without the lysosomal inhibitors, pepstatin A and E-64D.

Autophagy is an intracellular lysosomal clearance process associated with retinal degeneration and sphingolipid disorders [33–35]. As shown in Fig 7A, deletion of ASMase resulted in a 58±12.8% increase in LC3-II in the eyecups, suggesting the accumulation of autophagosomes in ASMase KO eyecups. However, LC3-I and beclin-1, the upstream regulators of autophagy signaling and crucial to the initiation of the formation of autophagosomes, showed no measureable difference between WT and ASMase KO eyecups. To determine if the autophagy disorder is a consequence caused by the ASMase deletion, we evaluated the levels of LC-3 isoforms in cultured cells, including a control fibroblast cell line (GM02226) and a Niemann-Pick type A dermal fibroblast cell line (NPD GM13205). As shown in Fig 7B, significant accumulation of LC-3II was observed in the NPD fibroblast cells, while no changes in LC3-I were observed. The accumulation of LC3-II could be caused by either the activation of autophagocytosis or by defects in the fusion process of autophagosomes with lysosomes [36]. To discriminate between these two possibilities, the cells were treated with two lysosomal inhibitors, pepstatin A and E-64D. The inhibitors increased the levels of LC3-II in the control cells, but not in the NPD cells. This result demonstrated that the fusion of autophagosome and lysosome was impaired in NPD cells.

## Discussion

ASMase is an essential enzyme for sphingolipid metabolism. The absence of ASMase expression in NPD individuals has been shown to lead to neurodegeneration in the brain [17]; however, studies in the retina are limited and have produced conflicting results. Examination of postmortem eyes from a NPD type A patient revealed abnormal storage inclusion bodies in various retinal cells [22]. In NPD type B patients, two independent studies showed phenotypes of vision loss in some patients [19, 23], while another study failed to identify any evidence of retinal degeneration in these individuals [20].

Our results demonstrated that age-dependent neural retinal degeneration occurs in the ASMase KO animals. However, the observation of normal retinal morphology and limited functional deficits at 1 month supports the idea that ASMase has a limited role, if any, in neural retinal development. However, additional studies are needed to definitively answer questions about the role of ASMase in retinal development and vascular function.

In young ASMase KO mice, the significant decrease of a-wave amplitude and eventual loss of photoreceptor outer segments and cell bodies is consistent with the idea that photoreceptor degeneration is a central feature of these animals. To investigate if additional outer retinal changes are expressed in these mice, ERG c-waves and RPE lipofuscin were evaluated. As shown in Fig 4, 1-month-old ASMase KO mice also exhibited significant reductions in c-wave amplitudes. Comparing the accumulation of lipofuscin in the RPE of to WT and ASMase^+/-^ mice, there was a dramatic elevation, with lipofuscin granules virtually filling the cytoplasm. The fluorescence emission spectra of the RPE granules are broadly similar to those reported previously [27]. The slightly broader emission spectrum of the ASMase KO granules (Fig 5D) may however indicate the presence of some differences in lipofuscin composition or environment in that case. The reduction in ERG c-wave amplitude demonstrated that in general RPE function is reduced, while the large accumulation of autofluorescent material was consistent with the idea that RPE lysosomal degradation is compromised in ASMase KO mice. Studies by Kim and colleagues [37] have shown that the process of RPE phagocytosis of outer segments utilizes autophagy-related mechanisms.

Autophagy is an intracellular recycling process involved in protein and organelle degradation via the lysosomal pathway. Studies have shown that autophagic activity decreases with age and more recently, altered autophagy has been implicated in retinal degeneration [35, 38–41]. Recent study has shown that ASMase KO mice have lysosomal damage and autophagy dysfunction in brain [42]. In the current study we demonstrated that the absence of ASMase in eyes from ASMase KO mice, or a fibroblast cell line from NPD type A patients, resulted in an increase in the LC3-II. Additionally, our study provided evidence that the increase in LC3-II results from the suppression of the fusion process of autophagosomes with lysosomes. These results supported the hypothesis that ASMase is required for autophagosome clearance, and in NPD patients this defect in the autophagy contributes to the development of this disorder. However, recent work by Toops and colleagues has shown that cholesterol-induced increases in ASMase activity can also reduce autophagy flux in the RPE and inhibiting ASMase activity could prevent this response [43]. While these results clearly link ASMase activity to autophagy in the RPE, understanding the biochemical mechanisms will require additional studies.

Finally, we observed an increase in levels of sphingosine, a known stress mediator, in the retinas of ASMase KO mice [11, 44]. As ASMase generates ceramide which is further broken down to sphingosine in the lysosome, the increase in sphingosine likely results from compensatory changes of other sphingolipid enzymes involved in maintaining normal levels of ceramides and other essential lipid-signaling molecules. Evidence for the existence of these compensating effects comes from our demonstration of normal ceramide levels in spite of the accumulation of SM in the retinas of the ASMase KO mice (Fig 6C). These results are consistent with the previous published studies, which showed that sphingomyelin but not ceramide levels are increased in ASMase knockout mice.[45, 46]. A compensatory increase in the activities of acid ceramidase may contribute to the increased sphingosine and dihydrosphingosine levels; however, understanding the precise mechanism underlying the elevation of these lipids will require further investigation.

In the present study we demonstrated that ASMase is necessary for the maintenance of normal retinal structure and function and defects in sphingolipid metabolism can lead to age-related retinal degeneration. These studies provided evidence that the absence of ASMase resulted in disrupted autophagy-related events in the retina, leading to the accumulation of lipofuscin material in the RPE and neural retina. As the accumulation of lipofuscin has been linked to retinal degenerations, ASMase KO mice may provide a novel model for studying how alteration in ASMase activity contributes to these processes.
